# Silent Obstruction: A Novel Complication of Portal Vein Thrombosis After Pancreatitis

**DOI:** 10.7759/cureus.88259

**Published:** 2025-07-18

**Authors:** Sneh Parekh, Ty J Merry, Vanshika Tripathi, Pramod Reddy

**Affiliations:** 1 Internal Medicine, University of Florida College of Medicine – Jacksonville, Jacksonville, USA

**Keywords:** anticoagulation, pancreatitis, pancreatitis complications, portal vein thrombosis, thrombosis, thrombotic events

## Abstract

Acute pancreatitis (AP) is a well-known cause of hospitalization and can lead to numerous complications. One such rare complication is portal vein thrombosis (PVT), defined as obstruction of the portal vein due to a thrombus within the vessel. The development of PVT in patients without any predisposing risk factors may pose a significant diagnostic challenge and can lead to delays in diagnosis and treatment. Here, we present the case of a patient with no past medical history and no significant risk factors, who insidiously developed PVT after initially being admitted with AP. The purpose of this case is to highlight this atypical presentation in the absence of risk factors and to emphasize the importance of repeat imaging in identifying PVT, allowing for rapid initiation of anticoagulation to improve long-term patient outcomes.

## Introduction

Acute pancreatitis (AP) is a well-known cause of gastrointestinal hospitalizations in the United States, with an estimated nearly 300,000 hospitalizations yearly [1]. As such, AP is the leading cause of hospitalization among gastrointestinal disorders in the United States [2]. Since pancreatitis is seen so commonly in the hospital setting, specific clinical criteria exist for its diagnosis of AP, and at least two of the three criteria must be present to confirm the diagnosis [2]. The pathophysiology of AP involves the premature activation of the enzyme trypsinogen to trypsin, initiating an inflammatory cascade that leads to widespread systemic inflammatory manifestations [3]. This can ultimately result in increased capillary permeability, endothelial damage, and microvascular thrombosis [3].

The treatment of AP focuses on supportive management and includes aggressive fluid resuscitation, pain management, and bowel rest. Further management involves addressing the local and systemic complications that may arise. Local complications include necrosis and pseudocysts, while systemic complications can include acute respiratory distress syndrome, third-spacing of interstitial fluid, thrombosis, and, in severe cases, sepsis, shock, and death [2,3].

One lesser-known complication of pancreatitis is portal vein thrombosis (PVT), which refers to obstruction of the hepatic portal vein due to a thrombus in the vessel. A retrospective study conducted between 2016 and 2019, involving over 1.3 million patients, demonstrated the rarity of PVT as an adverse outcome associated with AP, occurring in approximately 0.8% of cases [4]. Factors associated with PVT development include male sex, multiple (three or more) comorbidities, pseudocysts, along with sepsis, shock, and ileus [4]. Women had a 15% lower risk of developing PVT compared to men; patients with three or more comorbidities had an 87% higher risk of developing PVT than those without; and those with sepsis had a 55% higher risk of developing PVT [4]. Another retrospective study further demonstrated that all patients admitted with AP who developed splanchnic vein thrombosis were also associated with a form of severe pancreatitis, including pancreatic necrosis and peripancreatic collections [5]. The clinical manifestations of PVT in patients with AP are nonspecific and may include generalized abdominal pain, fever, nausea, vomiting, or abnormal laboratory findings. Finally, the incidence of mortality in AP patients with concomitant PVT was increased to 6.2%, compared to 2.18% in patients without PVT [4].

Given these nonspecific symptoms and the increased risk of mortality, the development of thrombosis in the form of PVT in patients without any additional predisposing risk factors may pose a significant diagnostic challenge, requiring early identification and treatment due to the high risk of adverse outcomes if not properly managed. While splanchnic vein thromboses are known complications of AP, PVT specifically is inherently rare, as described above, and is generally seen only in forms of severe AP. Additionally, due to the nonspecific symptoms associated with PVT, it remains a challenge to determine whether these general symptoms are part of the natural clinical course of AP or indicative of a newly developed PVT. As such, we aim to emphasize the importance of considering PVT in the differential diagnosis as a lesser-known complication of AP, one that can present even in the absence of risk factors and in otherwise non-severe cases of AP. Here, we present the case of a 40-year-old male with no past medical history and no other significant risk factors for PVT, apart from male sex, who insidiously developed PVT after initially being admitted to our hospital with a non-severe form of AP.

## Case presentation

**Figure 1:**
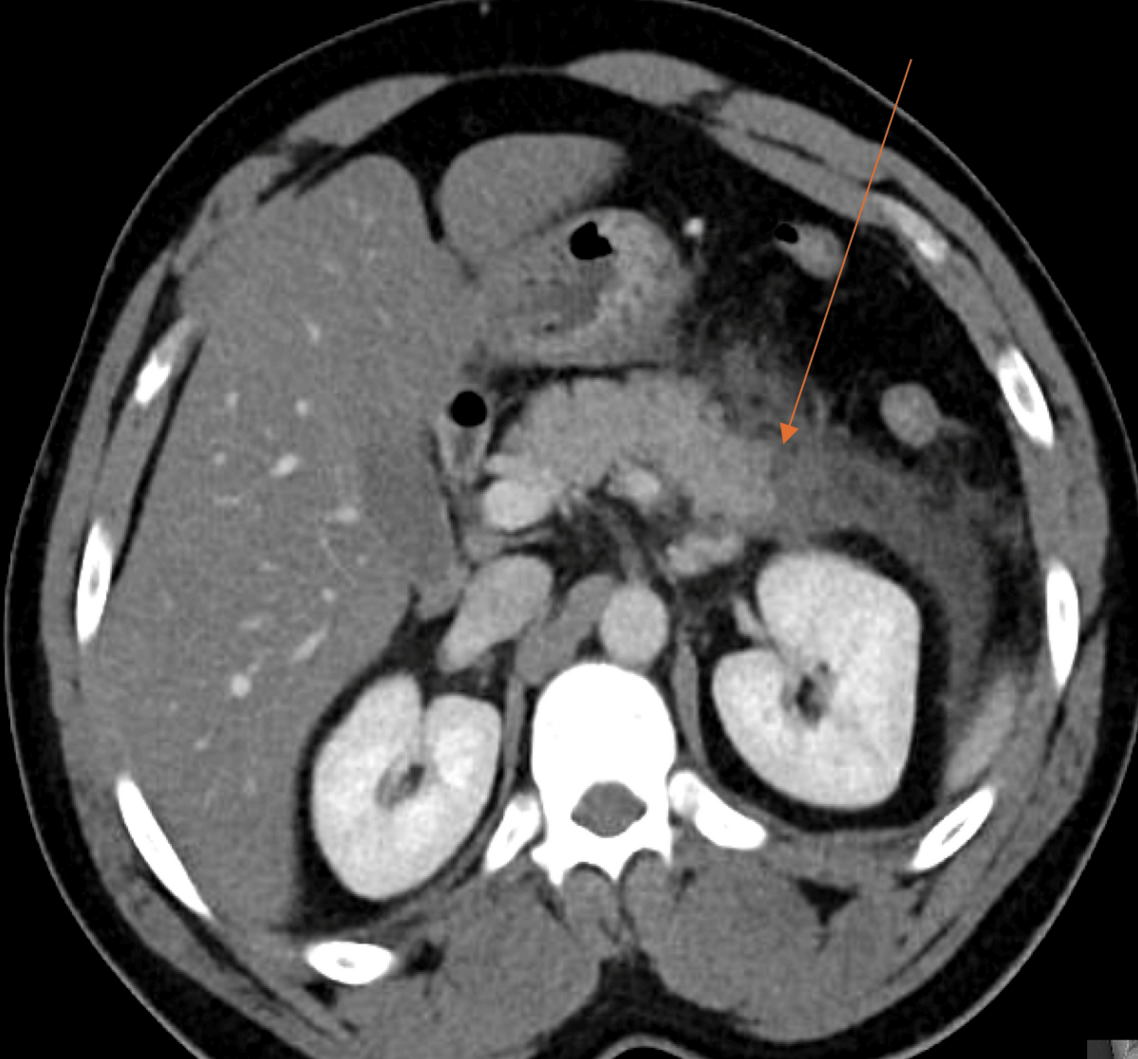
CT abdomen and pelvis showing acute interstitial edematous pancreatitis, obtained on admission, as indicated by the orange arrow CT: computed tomography

A 40-year-old male with no past medical history presented with sudden-onset epigastric abdominal pain that began the day prior, associated with nausea and vomiting. On presentation, vitals were significant for hypertension, tachycardia, and tachypnea. The physical examination revealed clear breath sounds bilaterally and normal heart sounds. The abdominal exam was notable for mild abdominal distention but showed no tenderness to light or deep palpation, and no guarding or rigidity. Laboratory testing was only significant for mild hyperkalemia of 6.1 mmol/L (reference: 3.4-4.5 mmol/L) in the setting of a repeatedly hemolyzed blood sample, and hypophosphatemia of 2.3 mg/dL (reference: 2.5-4.5 mg/dL), likely due to recent emesis with poor oral intake before presentation. Given high clinical suspicion, lipase was obtained and returned elevated at 384 U/L (reference: 0-60 U/L). Thereafter, imaging with computed tomography (CT) of the abdomen and pelvis showed acute interstitial edematous pancreatitis with surrounding nonlocalized fluid and stranding, with no evidence of drainable collections or abscesses (Figure 1).

Ranson’s score on admission was 1, indicating a low likelihood of severe AP and an estimated mortality of 1%. A lipid panel was also obtained, showing elevated total cholesterol at 233 mg/dL (reference: 100-199 mg/dL) and triglycerides at 831 mg/dL (reference: ≤150 mg/dL), potentially elucidating the underlying etiology of the patient’s pancreatitis. With the diagnosis of AP confirmed per clinical criteria (Table 1), having met all three criteria, the patient was medically managed with supportive care, including intravenous (IV) fluids, pain medications as needed, and bowel rest.

**Table 1:** Specific clinical criteria in the diagnosis of AP AP: acute pancreatitis; CT: computed tomography; MRI: magnetic resonance imaging; US: ultrasonography At least two of the three criteria must be present to make the diagnosis.

Diagnostic Criteria of AP
(1) Persistent and severe, acute-onset epigastric pain often radiating to the back
(2) Serum amylase or lipase ≥3 times the upper limit of normal
(3) Characteristic imaging findings of AP, seen on CT, MRI, or US

Within one day following admission, the patient reported improvement in his pain, and his diet was slowly advanced as tolerated, while also encouraging oral rehydration. The patient was started on atorvastatin 40 mg nightly and fenofibrate 108 mg daily for his hyperlipidemia.

However, later on hospital day one, the patient became febrile with a temperature of 101.5 °F and remained tachycardic on vital signs, with a heart rate reaching up to 140-150 beats/minute. Further evaluation in the form of two sets of blood cultures, a respiratory viral panel, and urinalysis returned negative for any source of infection; however, the patient was empirically started on piperacillin-tazobactam 4.5 mg every eight hours. He remained clinically stable; however, his severe hypertension and tachycardia would persist through his hospitalization, and he continued to have intermittent fevers, as seen in Table 2, despite reported resolution of his pain and a negative infectious workup, while also having initially been on empiric piperacillin-tazobactam. Due to concern for possible pancreatic necrosis and pseudocyst formation, on hospital day three, the patient was started on IV meropenem 1 g every eight hours for widened broad-spectrum empiric coverage.

**Table 2:** Daily vital signs throughout hospitalization min: minute; mmHg: millimeters of mercury Notably, the respiratory rate was not measured on days five and six due to stabilization throughout the hospitalization. Also, note the improvement in hemodynamic parameters on vital signs following the initiation of apixaban on day five.

Variables	Day 0 (Admission)	Day 1	Day 2	Day 3	Day 4	Day 5	Day 6 (Discharged)	Reference Ranges
Temperature (°F)	97.8	100.8 (would later increase to 101.5 by evening)	100.8	99.5	100.4	98.9	99.1	97.7–99.1
Heart rate (beats/min)	120	144	128	114	120	106	101	60–100
Respiratory rate (breaths/min)	29	18	20	18	18	---	---	12–20
Blood pressure (mmHg)	178/113	149/97	151/92	164/105	162/99	156/98	143/98	90/60–120/80

**Figure 2:**
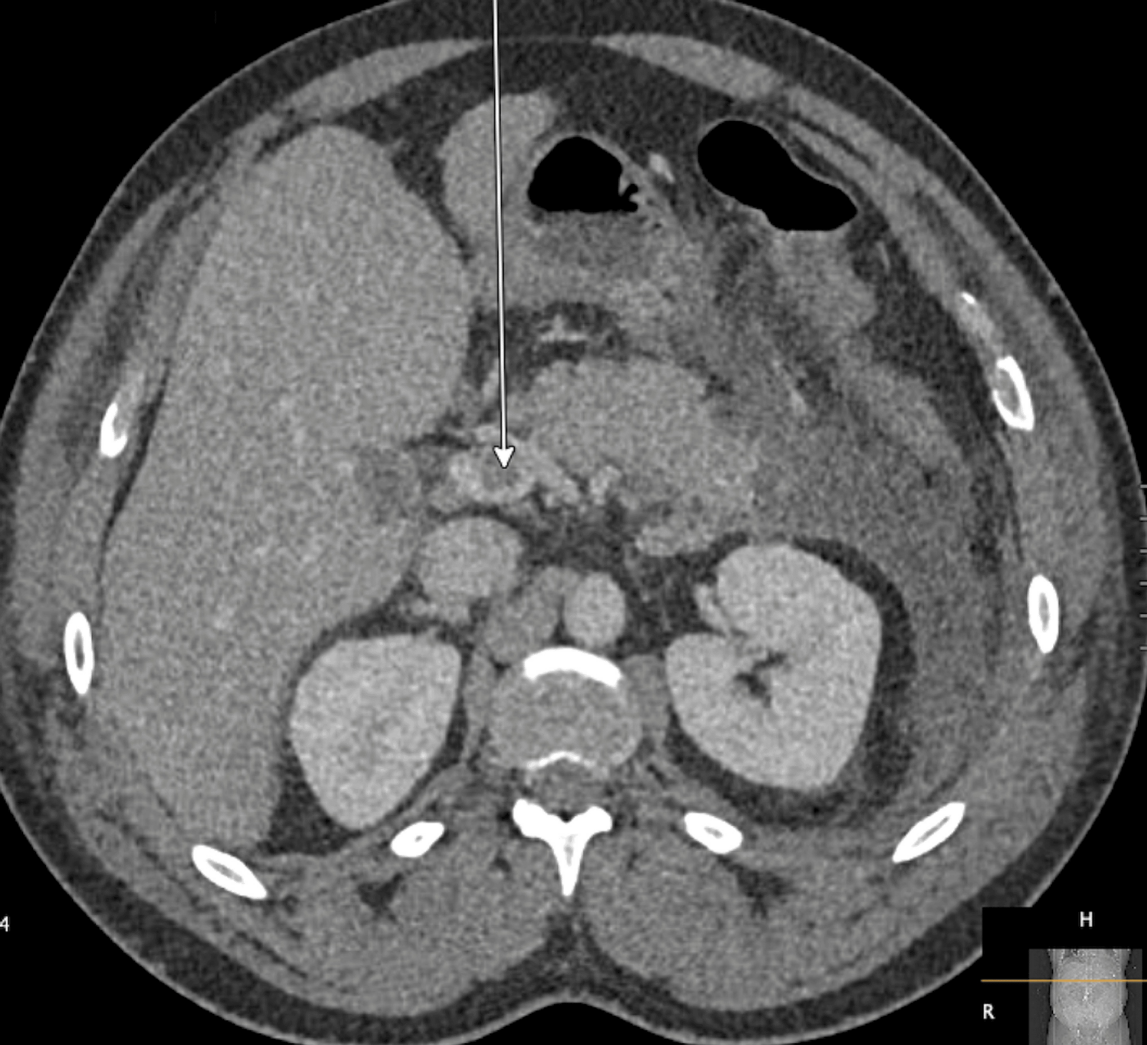
CT abdomen and pelvis with white arrow indicating the nonocclusive filling defect in the proximal portal vein, consistent with PVT CT: computed tomography; PVT: portal vein thrombosis

Due to concern for a local versus systemic complication of AP, a repeat CT abdomen and pelvis was obtained on hospital day four, demonstrating a new nonocclusive filling defect in the proximal portal vein. The patient was ultimately diagnosed with portal vein thrombosis (Figure 2).

Given the negative infectious workup and the absence of pancreatic necrosis, pseudocyst formation, or fluid collection, meropenem was deescalated to levofloxacin 750 mg daily to complete a seven-day course of empiric coverage. It is likely that the patient’s initial fevers and tachycardia were due to the inflammatory response triggered by AP. The patient remained clinically stable during his hospitalization and ultimately tolerated a full diet, with early resolution of pain. At that point, his vital signs would have been expected to normalize; however, the thrombotic event with PVT likely further exacerbated the fevers and tachycardia. As such, on hospital day five, the patient was also started on anticoagulation with apixaban following the diagnosis of the PVT. He was discharged on apixaban with instructions to complete a seven-day course at a loading dose of 10 mg twice daily, followed by a three-month course at 5 mg twice daily, along with instructions to follow up with his primary care provider.

## Discussion

AP is the leading cause of gastrointestinal hospital admissions in the United States [2]. Conservative management is the mainstay of treatment for AP, with adequate pain management, fluid resuscitation with IV fluids to correct hypovolemia and prevent pancreatic necrosis, and nutritional support, with advancement of diet as tolerated [6]. Nevertheless, even with appropriate management, complications such as peripancreatic fluid collections, pancreatic pseudocyst formation, and pancreatic necrosis may arise.

Among the complications of pancreatitis, vascular complications such as splenic and portal vein thromboses and pseudoaneurysm formation are also important to consider. PVT is a unique and rare complication of AP, only occurring in 0.8% of cases [4]. The pathophysiology involves inflammation from the underlying AP, leading to vascular injury and stasis, ultimately resulting in thrombus formation [7]. Research studies have shown that certain risk factors, including the presence of pancreatic pseudocysts, bacteremia, cirrhosis, male gender, and a history of previous venous thromboembolism, increase the likelihood of developing splanchnic vein thrombosis and PVT specifically [7]. A study conducted by Borbély et al. in 2024 also found an association between pancreatic necrosis and increased severity of AP, which can lead to an increased risk for the development of PVT [8]. In this case, apart from the patient’s male gender, no additional risk factors were present. Furthermore, the patient had a Ranson's score of one on admission, indicating a mild form of AP with an estimated 1% mortality risk, and, again, not fitting the severe classification of AP most often seen in literature to result in PVT [5,8].

PVT can present insidiously, resulting in worse patient outcomes if not identified and treated appropriately. Oftentimes, the nonspecific symptoms, including abdominal pain, nausea, and fever seen in PVT, can overlap with those of AP and other local and systemic complications of AP, making the diagnosis of PVT particularly challenging [5]. The presence of fevers and persistent tachycardia in our patient, in the absence of an obvious infection, along with a negative infectious workup, prompted repeat imaging, after which PVT was identified. Contrast-enhanced CT is the investigational modality of choice for evaluation of all splanchnic vein thromboses, and clinicians should keep PVT high on their differential [9]. Finally, our patient was started on therapeutic anticoagulation with apixaban. Per the literature, there is little consensus on the role of therapeutic anticoagulation in splanchnic vein thrombosis patients in the setting of AP, with an individualized patient-to-patient approach required [10]. Given our patient's persistent fevers and tachycardia, and lack of past medical history that would indicate a high risk for bleeding complications, it was necessary to treat our patient's PVT with the appropriate anticoagulation in the form of apixaban.

While AP is conservatively managed with supportive care, such complications, as in the case of our patient, require prompt attention, investigation, and further work-up for causative sources, due to the risk for mortality. In the absence of obvious risk factors, identifying key details in the patient’s hemodynamic status, while ruling out an infectious etiology, was valuable in raising the index of suspicion of a complicated AP, warranting repeat abdominal imaging to diagnose PVT. In doing so, it ultimately helped guide the appropriate management and allowed for rapid initiation of anticoagulation for treatment.

## Conclusions

This case highlights the importance of considering PVT as a complication of AP in patients without obvious risk factors. PVT may present without any distinct clinical manifestations, often masquerading under the same symptoms as AP, and in the absence of risk factors. In patients with AP who develop hemodynamic instability with fever, tachycardia, and hypertension/hypotension, there should be a low threshold for obtaining repeat abdominal imaging, particularly CT scans, to identify major post-pancreatitis sequelae. PVT is one such rare complication, and correctly identifying PVT in an atypical presentation can allow for rapid initiation of anticoagulation and improve long-term patient outcomes.
